# Differential Roles of the Two Raphe Nuclei in Amiable Social Behavior and Aggression – An Optogenetic Study

**DOI:** 10.3389/fnbeh.2018.00163

**Published:** 2018-08-02

**Authors:** Diána Balázsfi, Dóra Zelena, Kornél Demeter, Christina Miskolczi, Zoltán K. Varga, Ádám Nagyváradi, Gábor Nyíri, Csaba Cserép, Mária Baranyi, Beáta Sperlágh, József Haller

**Affiliations:** ^1^Laboratory of Behavioural and Stress Studies, Institute of Experimental Medicine, Hungarian Academy of Sciences, Budapest, Hungary; ^2^János Szentágothai School of Neurosciences, Semmelweis University, Budapest, Hungary; ^3^Laboratory of Cerebral Cortex Research, Institute of Experimental Medicine, Hungarian Academy of Sciences, Budapest, Hungary; ^4^Laboratory of Molecular Pharmacology, Institute of Experimental Medicine, Hungarian Academy of Sciences, Budapest, Hungary; ^5^Institute of Behavioural Sciences and Law Enforcement, National University of Public Service, Budapest, Hungary

**Keywords:** aggression, serotonin, glutamate, GABA, dorsal raphe, median raphe

## Abstract

Serotonergic mechanisms hosted by raphe nuclei have important roles in affiliative and agonistic behaviors but the separate roles of the two nuclei are poorly understood. Here we studied the roles of the dorsal (DR) and median raphe region (MRR) in aggression by optogenetically stimulating the two nuclei. Mice received three 3 min-long stimulations, which were separated by non-stimulation periods of 3 min. The stimulation of the MRR decreased aggression in a phasic-like manner. Effects were rapidly expressed during stimulations, and vanished similarly fast when stimulations were halted. No carryover effects were observed in the subsequent three trials performed at 2-day intervals. No effects on social behaviors were observed. By contrast, DR stimulation rapidly and tonically promoted social behaviors: effects were present during both the stimulation and non-stimulation periods of intermittent stimulations. Aggressive behaviors were marginally diminished by acute DR stimulations, but repeated stimulations administered over 8 days considerably decreased aggression even in the absence of concurrent stimulations, indicating the emergence of carryover effects. No such effects were observed in the case of social behaviors. We also investigated stimulation-induced neurotransmitter release in the prefrontal cortex, a major site of aggression control. MRR stimulation rapidly but transiently increased serotonin release, and induced a lasting increase in glutamate levels. DR stimulation had no effect on glutamate, but elicited a lasting increase of serotonin release. Prefrontal serotonin levels remained elevated for at least 2 h subsequent to DR stimulations. The stimulation of both nuclei increased GABA release rapidly and transiently. Thus, differential behavioral effects of the two raphe nuclei were associated with differences in their neurotransmission profiles. These findings reveal a surprisingly strong behavioral task division between the two raphe nuclei, which was associated with a nucleus-specific neurotransmitter release in the prefrontal cortex.

## Introduction

Early studies from the 70’s indicated that the serotonergic system plays an important role in aggression control. These studies revealed that the destruction of the main raphe nuclei (dorsal raphe, DR; median raphe region, MRR) increase aggression in mice, serotonin depletion by systemic para-chlorophenylalanine facilitates non-specific killing behavior in rats, and that aggressive behavior in humans is associated with low serotonin levels, the behavior being reversed by serotonin-enhancing drugs (Kostowski et al., 1975; Miczek et al., 1975; Greenberg and Coleman, 1976). The role of serotonin in aggression control was confirmed by subsequent animal and human studies. It was even stated that serotonin is the primary determinant of inter-male aggression, other neurotransmitters affecting it indirectly via serotonin signaling (Nelson and Chiavegatto, 2001). Besides controlling natural manifestations of aggressive behavior (Rosell and Siever, 2015; Sandi and Haller, 2015) deficits in serotonergic neurotransmission are implicated in the development of abnormal animal aggression, i.e., those aggressions that overpass species-specific levels and behavioral patterns (Haller et al., 2005; Haller et al., 2014; Miczek et al., 2015; Sandi and Haller, 2015). Not surprisingly, it was suggested that laboratory research aiming at the development of new psychotropic drugs for the treatment of aggression problems should target the serotonergic system (Olivier, 2015). Research performed in primates and humans support these findings obtained mainly in rodents, including the use of serotonergic compounds for the treatment of aggression-related psychopathologies (Coccaro et al., 2015; de Almeida et al., 2015; Glick, 2015; Zhang-James and Faraone, 2016). However, findings on the role of serotonin in aggression control are in many respects conflicting. Laboratory studies showed for instance that the chronic pharmacological reduction of serotonin availability by a series of serotonergic compounds promoted aggression, but aggression decreased when serotonin release was inhibited acutely (de Boer and Koolhaas, 2005). Some clinical studies show that selective serotonin reuptake inhibitors (SSRIs), decrease aggression in certain aggression-related psychopathologies while being ineffective in others (Coccaro et al., 2015; Glick, 2015); moreover, SSRIs promoted rather than decreased aggression in a series of well documented cases (Bielefeldt et al., 2016; Sharma et al., 2016). The reasons of such discrepant findings are largely unknown.

One possible explanation may reside in the differential involvement of the two main serotonergic nuclei in aggression control, e.g., the MRR and DR. These raphe nuclei send parallel and overlapping projections to limbic structures including the cortex in both animals and humans, but their projection patterns differ, and differences were found with regard to their functional and structural characteristics, including their sensitivity to psychoactive agents (Mulligan and Tork, 1988; Wilson and Molliver, 1991; Hornung, 2003; Hensler, 2006). Perhaps the largest difference between the two nuclei is that the majority of axons originating from the MRR form synapses in the forebrain, whereas DR projections rarely form synapses and exert their effects via volume transmission (Hornung, 2003; Hensler, 2006). Volume transmission (or non-synaptic communication) is typical to monoamine (particularly serotonergic and noradrenergic) and peptidergic neurotransmission. It affects extended brain areas, and targets high-affinity receptors located on extra-synaptic sites, e.g., the soma or dendrites of neurons, and modulate neuron activity rather than transmit information in the way synaptic communication does (Vizi, 2000; Leng and Ludwig, 2008). The findings briefly reviewed above show that the projections of the two raphe nuclei have different anatomical and functional properties; consequently, they may have distinct roles in behavioral control.

To investigate this issue, here we studied the behavioral consequences of MRR and DR stimulation on the social and aggressive behaviors of mice (i.e., non-aggressive and aggressive social interactions, respectively). Stimulations were performed by optogenetic techniques that allow a more precise control over the stimulated brain areas than electric stimulations. Several studies have shown that raphe nuclei are not homogenous neurochemically (Moore, 1980). Therefore, we also studied the impact of stimulations on neurotransmitter release in the prefrontal cortex, a major site of aggression control. In addition to serotonin release, we studied the release of glutamate and GABA, which are expressed by a large share of raphe neurons (Commons, 2009; Jackson et al., 2009; Varga et al., 2009; Sos et al., 2017); moreover, glutamate is often co-expressed with serotonin in the very same raphe neurons (Shutoh et al., 2008; Gagnon and Parent, 2014). We hypothesized that the DR and MRR are different in terms of both behavioral and neurochemical effects.

## Materials and Methods

### Animals

**FIGURE 1:**
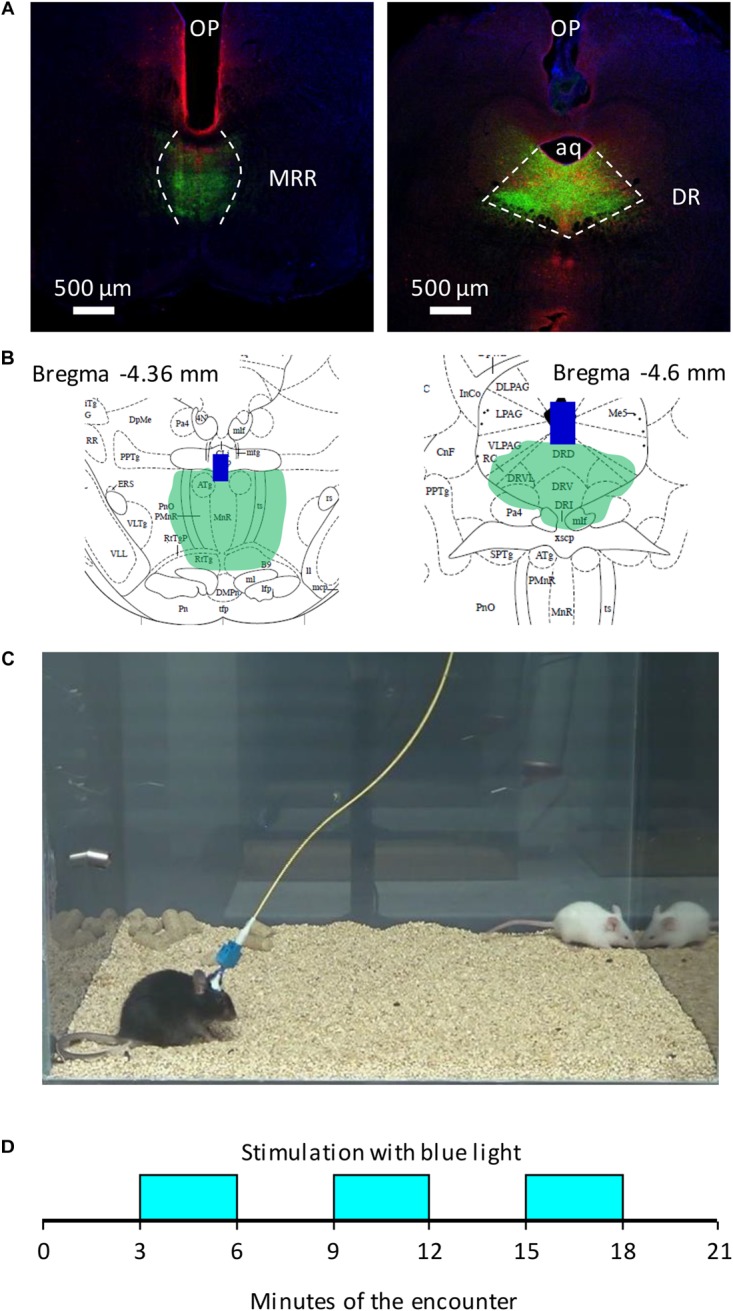
Schematic representation of behavioral studies. **(A)** Representative photomicrographs of the placement of optic fibers and the extent of ChR2 expression in the brainstem. **(B)** Schematic representation of optic fibers and ChR2 expression on Paxinos (2001) plates in two mice; **(C)** the cage of behavioral studies, with an experimental (black, Bl6), and a stimulus mouse (white, CD1); **(D)** the timing of optogenetic stimulations. *AQ*, aqueduct; *blue bars* **(B)**, the tip of optic fibres; *DR*, dorsal raphe; *green area* **(A,B)**, virus labeling; *MRR*, median raphe region; *OP*, optic fibers; *red immunochemical labeling* **(A)**, serotonergic neurons.

Adult C57BL/6N male mice (Charles River, Budapest, Hungary), were used as residents in social encounters. They were 12–14 weeks old at the beginning of the study, e.g., at the time of their surgery. We used 20–25 days old CD1 mice (Charles River, Budapest, Hungary) as opponents in social interaction tests (**Figure 1C**). Animals were housed individually under a standard 12 h light–dark cycle (lights on at 6 am), with food and water available *ad libitum*. Experiments were approved by the local committee for animal health and care (Animal Welfare Committee of the Institute of Experimental Medicine) and performed according to the European Communities Council Directive recommendations for the care and use of laboratory animals (2010/63/EU).

### Virus Injection and Optogenetics

For the optical control of raphe regions, 40 nL adeno-associated virus vector (AAV; Penn Vector Core, PA, United States) encoding ChR2 (AAV2.5.hSyn.hChR2(H134R)eYFP.WPRE.hGH; 1.3e12 GC/ml; Addgene26973) were injected into the median raphe region (MRR) or dorsal raphe (DR) from glass pipettes (tip diameter 20–30 μm) connected to a MicroSyringe Pump Controller (World Precision Instruments, Sarasota, FL, United States) under deep anesthesia (intraperitoneal injection of 25 mg/kg xylazine and 125 mg/kg ketamine in 0.9% NaCl) (Balazsfi et al., 2017). The coordinates of the virus injection were the followings: MRR: AP: -4.10 mm, L: 0.0 mm, DV: 4.60 mm; DR: AP: -4.40 mm, L: 0.0 mm, DV: -3.40 mm. Two weeks after the injection mice were implanted with optic fibers (core diameter: 105 μm; flat tip; MRR: 10° from dorsal, AP: -4.80 mm, L: 0.0 mm, DV: -4.10 mm; DR: 10° from dorsal, AP: -5.20 mm, L: 0.0 mm, DV: -3.35 mm). Optic fibers for implantation and light stimulation were custom made from multimode optical fiber (AFS 105/125Y, NA: 0,22, low-OH, Thorlabs Corp., Munich, Germany) and flanged zirconia ferrule (LMFL-172-FL-C35-OSK, Senko, Hampsire, United Kingdom). Implants were secured by screws and acrylic resin (Duracryl Plus; SpofaDental, Czech Republic). Behavioral experiments started after 4–7 days recovery. Laser beams (473 nm) were generated by low noise diode-pumped solid-state lasers (Ikecool Corp., Anaheim, CA, United States), then collimated and guided to the implanted optic fiber by fiber-optic patch cords (FT900SM and FT030-BLUE, Thorlabs Corp.). Net energy output was measured by laser power meter (Coherent, LaserCheck, Santa Clara, CA, United States) before and after the experiments. Data were used only when optic fibers transferred 10-20 mW net energy at continuous light emission. The frequency of optogenetic stimulation was 20 Hz (25 ms pulses) in both the behavior and the microdialysis study.

### Experimental Design

**FIGURE 2:**
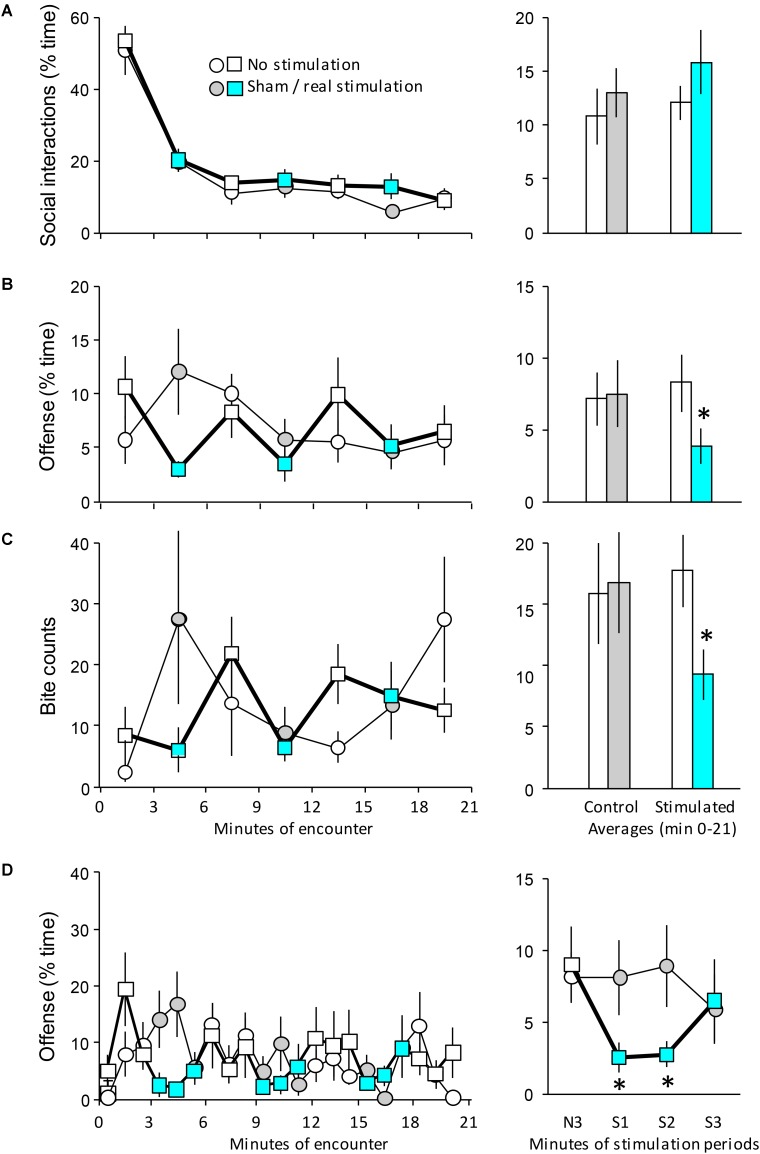
The behavioral effects of the optogenetic stimulation of the MRR on day 1. Mice implanted with optic fibers encountered an unfamiliar opponent in a neutral arena (see **Figure 1C**). **(A–C)** *Left-hand panels*: the duration of behaviors in 3-min bins; *right-hand panels*: the average duration of behaviors (0–21 min). **(D)** *left-hand panel*: the duration of offensive behaviors shown on a minute-by-minute basis; *right-hand panel*: effect of stimulation on the duration of offense. Values represent the average of the three stimulations shown on the left-hand graph of the same panel. *N3*, the average of the last (3rd) minutes of the non-stimulated phase that preceded stimulations; *S1-3*, averages of the 1st, 2nd, and 3rd min of the stimulation periods. The timing of stimulation was indicated by the color code; circles indicate controls, squares and bold lines indicate stimulated mice. Sample sizes: non-stimulated *n* = 8; stimulated *n* = 9. ^∗^Significant effect of optic stimulation in *post hoc* tests (*p* < 0.05 at least). Note that there were multiple significant differences between the time-points of left-hand panels; for clarity, the significance of such differences was shown on right-hand panels only.

**FIGURE 3:**
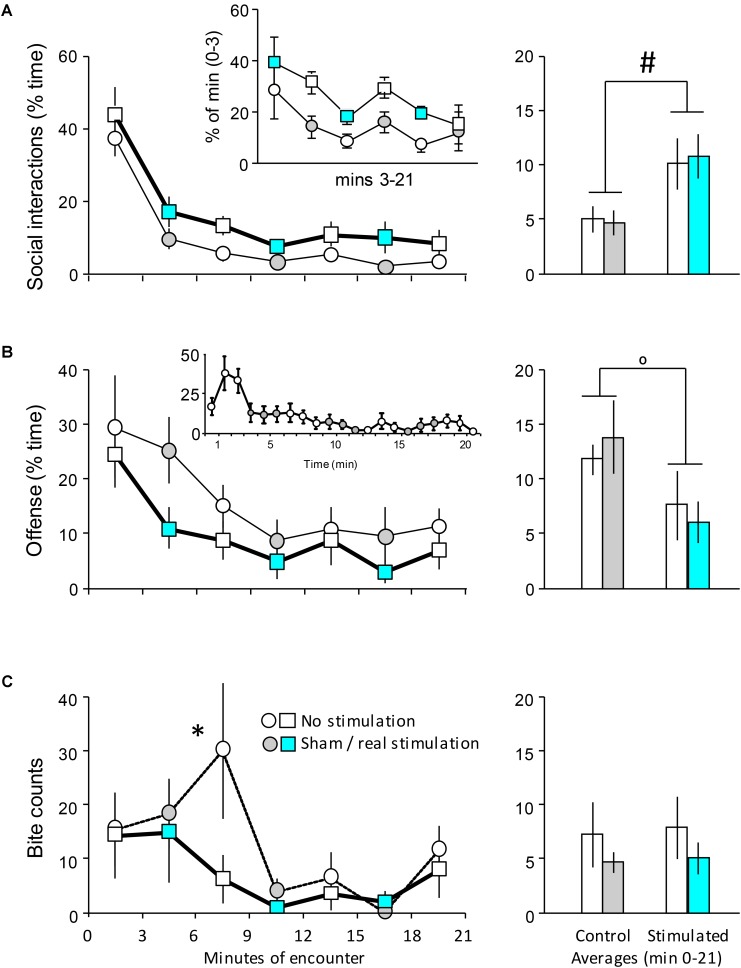
The behavioral effects of the optogenetic stimulation of the DR on day 1. Mice implanted with optic fibers encountered an unfamiliar opponent in a neutral arena (see **Figure 1C**). **(A–C)** *left-hand panels*: the duration of behaviors in 3-min bins; *right-hand panels*: the average duration of behaviors (min 0–21). Insert of **(A)**, values expressed as percent of min 0–3. See text for explanations. Insert of **(B)**, min-by-min representation of the duration of offense in controls (see text). The timing of stimulation was indicated by the color code; circles indicate controls, squares and bold lines indicate stimulated mice. Sample sizes: non-stimulated *n* = 7; stimulated *n* = 7. ^∗^Significant difference between sham and real stimulation in *post hoc* tests; ^#^Significant main effect (*p* < 0.05 at least in both cases); ^o^Trend-level main effect (0.1 < *p* < 0.05).

Mice were exposed at 2-day intervals to four social interaction tests (see below); i.e., the total duration of the study (including inter-trial days) was 8 days. We used a roman square design. On day one, half of the animals were stimulated and half served as control. Controls were sham stimulated, i.e., they were connected to optic fibers but light was not delivered. The effects of optic stimulation were studied on this experimental day, when all mice were experimentally naïve. The findings of this trial were shown in **Figure 2** (MRR stimulation) and **Figure 3** (DR stimulation).

**FIGURE 4:**
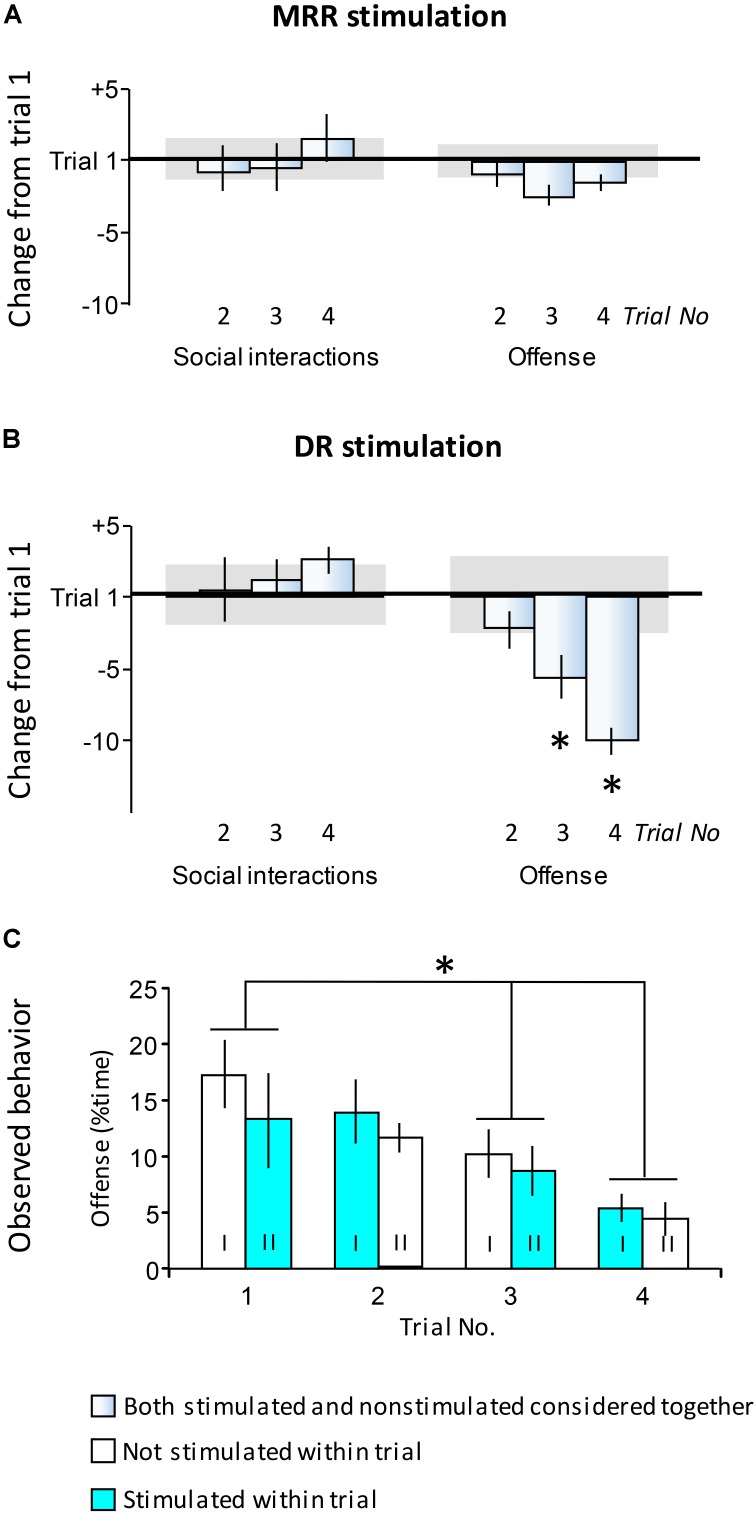
Carryover effects of stimulations. Findings presented here show behavior observed in trials 2, 3, and 4 when all mice had a history of stimulation. The aim of this study was to investigate carryover effects (see Experimental design). **(A,B)** Differences in the duration of behaviors as compared to trial 1. Values show differences in the time devoted to a particular behavior expressed as the percentage of total test time. **(C)** The duration of offense in trials 1–4. Here data were shown separately for mice stimulated or non-stimulated within the particular trials. Each cohort of mice (indicated by roman numbers) was submitted to alternating trials of stimulation and non-stimulation. *Horizontal line in* **(A,B)**, the average duration of behaviors in trial 1; *Horizontal bars in* **(A,B)** standard errors of the average duration of behaviors in trial 1; *DR*, dorsal raphe; *MRR*, median raphe region; ^∗^Significant differences between trials in *post hoc* tests (*p* < 0.05 at least).

Three additional social encounters were run to investigate the carryover effects of stimulation. On each of these days, treatments were reversed compared to the previous trial such that each animal was exposed to a total of two control, and two stimulated social interaction tests. By carryover effects, we mean here those effects of stimulation that are detectable on the subsequent, non-stimulation trial. The findings of these trials were shown in **Figure 4**, and were expressed as changes compared to day 1. It was hypothesized that carryover effects, if present, would be independent of the ongoing stimulation. Therefore, the actual stimulation status of mice was not considered when carryover effects were studied. Note that there was no significant interaction between time and stimulation in trials 2–4.

A separate group of mice was used in the *in vivo* microdialysis study (see below).

### Social Interaction Test

The implanted animal (resident) was equipped with an optic fiber and was placed into the test cage (29 cm × 35 cm × 40 cm) with water and food available *ad libitum* for a 30 min habituation period. The test started when the intruder (CD1 mouse) was placed into the same cage (**Figure 1C**). The test lasted 21 min and divided to 3 min periods (**Figure 1D**). The 20 Hz optogenetic stimulation was administered in the second (3–6 min), fourth (9–12 min) and sixth (15–18 min) periods or the mice were left for 21 min with the intruder without stimulation.

We videotaped and later scored the behavior of resident (experimental) mice by means of a computer-based event-recorder software^1^. The experimenter was blind to treatments. We recorded the following behaviors: *inactivity/resting* (no obvious activity), *exploration/walking* (walking through the cage or sniffing directed toward the environment), *social investigation* (sniffing at partner or anogenital sniffing), *aggressive grooming* (pushing down the opponent, while it is standing or trying to escape, nibbling the fur and the skin with quick movements of the head), *tail rattling* (rapid rattling of the tail while the subject faces its opponent), *wrestling* (wrestling movements often associated with biting), *chasing* (quickly following the opponent which is fleeing; this behavior was subsequent to the delivery of bites to the opponent), *defensive upright* (trials of keeping the opponent at distance with forepaws while rising on hind legs), *avoidance* (evading the approaching opponent), and *flight* (quickly moving away from the chasing opponent). Defensive behaviors (defensive upright, avoidance, and flight) were extremely rare, whereas resting and exploration did not differentiate the groups. Therefore, these behaviors were not shown. We summed up aggressive grooming, tail rattling, wrestling and chasing as offensive behaviors. We recorded both the duration and frequency of all behaviors. For offense, we showed durations only, because frequencies and durations were highly correlated. In the case of bites, we showed frequencies, because these were very brief, and frequencies characterized them better than durations.

### *In Vivo* Microdialysis

**FIGURE 5:**
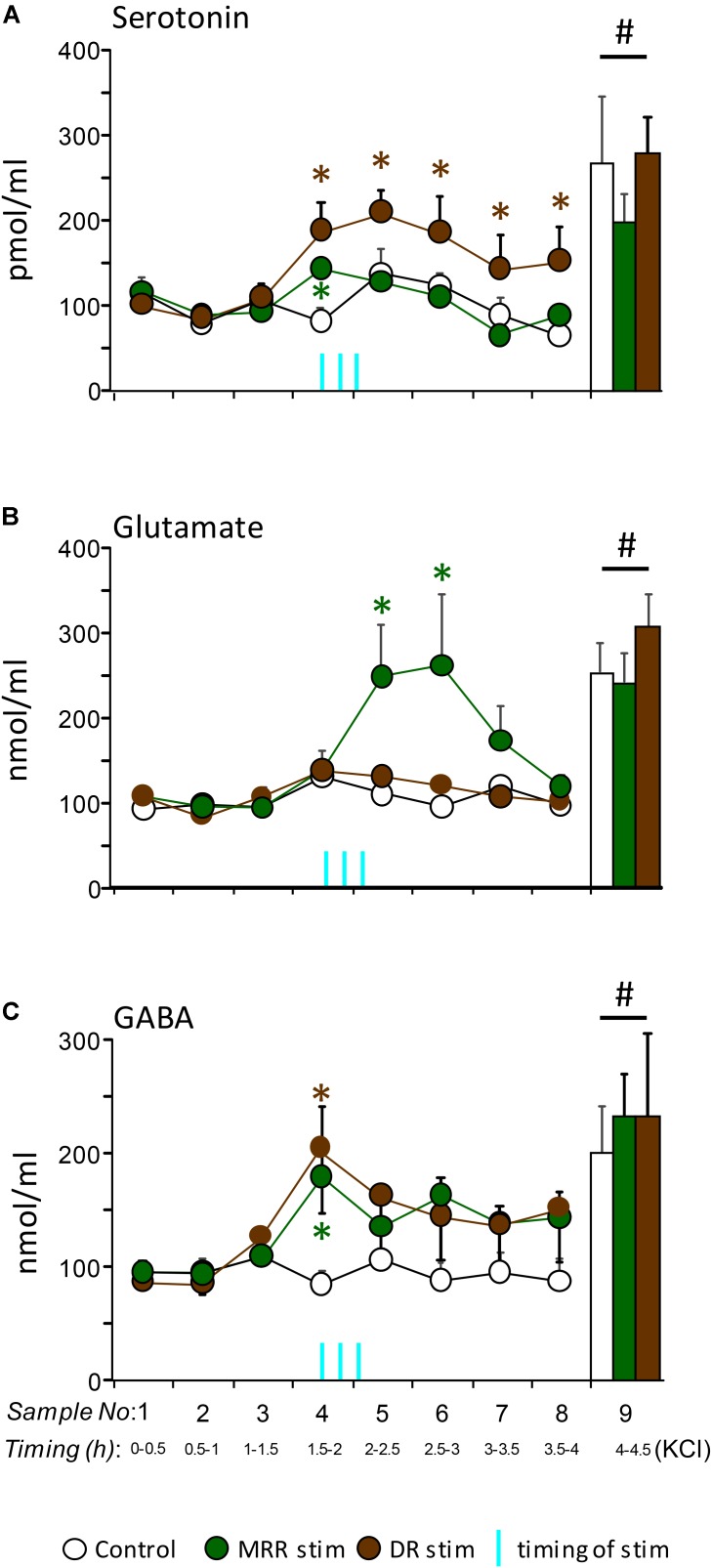
*In vivo* release of serotonin **(A)**, glutamate **(B)**, and GABA **(C)** in the prefrontal cortex of mice stimulated optogenetically in their raphes (median raphe region, MRR; dorsal raphe, DR). The stimulation protocol was identical with that employed for behavioral studies. *Vertical blue lines*, the timing of stimulations. Note that the first stimulation was started 90 min after the last basal sampling and 15 min before the fourth sampling, whereas the third stimulations started right at the beginning of the fifth fraction. Sample sizes: control *n* = 6; MRR stimulation *n* = 9; DR stimulation *n* = 5. *Vertical columns at the right-hand side of graphs*, neurotransmitter responses to the infusion of KCl into the raphes. *DR*, dorsal raphe; *MRR*, median raphe region; ^∗^significant effect of stimulations compared to control levels, same time-point; ^#^significant effect of KCl infusion as compared to baseline levels (the first three time points of each curve).

Eight weeks after AAV-ChR2 injection mice were implanted with the optic fiber as described above. After 4–7 days recovery the animals were anesthetized by intraperitoneal 20% urethane (Reanal; Budapest, Hungary) and microdialysis probe [EICOM CX-I Brain Probe (membrane: artificial cellulose, molecular weight cut off: 50,000 Da, OD: 0.22 mm, length: 2 mm)] was inserted into the prefrontal cortex (AP: -4.80 mm; L: 0.0 mm; DV: 5.50 mm), while optic fiber was connected to MRR or DR region. After 2 h equilibration period we collected 9 samples, one in every 30 min. Perfusion rate was 2 μl/min (**Figure 5**) (Goloncser et al., 2017). The first three samples served as baseline. Stimulation started 15 min before the end of the fourth sampling to detect rapid responses. The last stimulation started at the beginning of the fifth sampling period to investigate the habituation of neurotransmitter release to repeated stimulations. The stimulation protocol was identical with that shown in **Figure 1D**. We continued sampling for an additional 1.5 h (samples 6–8). The last sample was collected during the administration of 100 mM KCl for 5 min. This was performed to test the responsiveness of neurons.

### HPLC Analysis of Neurotransmitters

#### Neurotransmitters Serotonin, Glutamate and GABA in Dialysates Were Determined by Using HPLC

Method (Goloncser et al., 2017). The extraction solution (PCA) was 0.1 M perchloric acid that contained theophylline (as an internal standard) at 10 mM concentration. Initial volume of dialysis samples was measured and then diluted with an equal volume of ice cold PCA then supplemented with mobile phase “A” to 300 μL. The sample was centrifuged at 3510 *g* for 10 min at 0–4°C and 240 μL was injected onto the enrichment column. The remainder (60 μL) of the microdialysis sample was diluted with distilled water and the pH was adjusted to 10.5 with 2.7 M Na_2_CO_3_. The samples were reacted with (20 μL) 20 mM dansyl chloride for 15 min at 70° temperature than the reaction was stopped by 10 μL formic acid. To determine glutamate and GABA content, the volume of 350 μL of the reaction mixture was injected onto the enrichment column.

The levels of serotonin were determined by online column switching separation using Discovery HS C18 50 × 2-mm and 150 × 2-mm columns. The flow rate of the mobile phases [“A” 10 mM potassium phosphate, 0.25 mM EDTA “B” with 0.45 mM octane sulphonyl acid sodium salt, 8% acetonitrile (v/v), 2% methanol (v/v), pH 5.2] was 350 or 450 μl/min, respectively in a step gradient application. The enrichment and stripping flow rate of buffer [10 mM potassium phosphate, pH 5.2] was 4 min. The total runtime was 55 min. The HPLC system used was a Shimadzu LC-20 AD Analytical & Measuring Instruments System, with an Agilent 1100 Series Variable Wavelength Detector set at 253 nm and an electrochemical (EC) amperometric detector BAS 400, Bioanalytical System set at 730 mV potential.

The levels of dansylated amino acids (glutamate and GABA) were separated by the above column system. The flow rate of mobile phases [“A” 10mM ammonium formate, 16.8% acetonitrile (v/v), methanol 4.8% (v/v), “B” 10 mM ammonium formate, 70% acetonitrile (v/v), methanol 20% (v/v), pH 3] was 400 μl/min in a linear gradient mode. The enrichment and stripping flow rate of the buffer [10 mM ammonium formate, 1.9% acetonitrile (v/v), 1.1% methanol (v/v)] was 300 μL/min during 4 min and the total runtime was 55 min. The used analytical system was the above, Shimadzu LC-20 System, with Gilson Model 121 Fluorimeter set at 340 nm excitation and 450 nm emission wavelength.

The recovery of the implanted microdialysis probes was evaluated at the end of experiment. The *in vitro* extraction efficiency for serotonin, glutamate and GABA were estimated to be 21.1 ± 4.8%, 17.1 ± 2.8%, and 21.9 ± 3.4%, respectively. The concentrations of serotonin, glutamate and GABA were expressed in percentage (mean ± SEM) of baseline concentrations in order to monitor changes from basal levels after optical stimulation.

### Anatomical Analysis

After termination of the behavioral experiments mice were deeply anesthetized (see above) and transcardially perfused with 0.1M phosphate buffered saline (PBS) for 1 min, then with 4% (w/v) paraformaldehyde (PFA) in PBS for 20 min. Optic fibers were carefully removed, brains were taken out, and post-fixed for 24 h in fixative at +4°C. Brains were cryo-protected by 20% glucose-PBS solution for 24 h at +4°C. At the end of the microdialysis optic fiber and microdialysis probe were removed carefully, and brains were postfixed for 24 h in 30% glucose containing PFA at +4°C. To enhance the green fluorescence protein (GFP) signal and to facilitate the identification of the MRR and DR, immunofluorescent staining was carried out on 50-μm-thick coronal sections (prepared on a Vibratome VT1200S, Leica, Wetzlar, Germany). Primary antibodies were diluted in Tris-buffered saline (TBS) (Rabbit-anti-Serotonin, 1:10000, ImmunoStar, Hudson, WI, United States; CatNo: 20080; Chicken-anti-GFP, 1:2000, Life Technologies, Carlsbad, CA, United States; CatNo: A10262) and were incubated for 2 days. After washing, sections were incubated in secondary antibody solution overnight (Cy3-conjugated Donkey-anti-Rabbit, 1:500, Jackson ImmunoResearch West Grove, PA, United States; CodeNo:711-165-152; Alexa488-conjugated Goat-anti-Chicken, 1:1000, Life Technologies, CatNo: A-11039; diluted in TBS). After multiple washes, sections were mounted and were evaluated with a Zeiss Axioplan microscope, and images were taken with an Olympus DP70 camera.

The position of the tip of the optical fiber, microdialysis probe and the size of the virus infected area were determined on micrographs by using on overlay of the stereotaxic atlas images on the series of images of the MRR and DR (Paxinos, 2001) (**Figures 1A,B**). We estimated the laser-illuminated volume based on the measurements by Yizhar et al. (2011). Mice with weak virus expression in the MRR or DR or with the optic fiber outside these regions, or the microdialysis probe outside the PFC were excluded from the analysis.

### Statistics

Data were represented as means ± standard error of the mean. Behavioral differences were evaluated by repeated measure ANOVA when temporal data series were evaluated. Two-way ANOVA was performed when the individual time-points of such temporal data series were averaged (see for instance the right-hand panels of **Figure 2**). Factors were indicated in Results. ANOVA was followed by Dunnet *post hoc* comparisons where main effects were significant. In the case of bite counts, which did not fulfill ANOVA requirements, statistical differences were evaluated by the median test, a subtype of Pearson’s chi-squared test. In the *in vivo* microdialysis study, a two factor repeated measures ANOVA was employed (repeated measures factor 1 was ‘time’; factor 2 was ‘groups’). P values lower than 0.05 were considered significant; *P*-values lower than 0.1 but larger than 0.05 were identified as trends.

## Results

### Acute Effects of Intermittent MRR Optic Stimulation

Social interactions were intense during min 0–3 of the 21 min-long encounter in controls, but decreased rapidly and were maintained at low levels throughout the encounter [*F*_time_(6,90) = 69.14; *p* < 0.0001] (**Figure 2A**). The optic stimulation of the MRR did not affect social behavior [*F*_treatment_(1,15) = 0.42; *p* > 0.6; *F*_interaction_(6,90) = 0.41; *p* > 0.9]. In sharp contrast, MRR stimulation strongly influenced aggressive interactions. In controls, aggressive interactions showed low levels in the first 3 min of the encounter, reached a peak between min 3 and 6, and gradually decreased thereafter (**Figure 2B**, circles). Changes in MRR-stimulated mice did not follow this pattern (**Figure 2B**, squares). Values comparable with those seen in controls were recorded in the periods when stimulations were not administered, but the duration of aggressive interactions sharply decreased during stimulation periods [*F*_treatment_(1,15) = 0.03; *p* > 0.9; *F*_time_(6,90) = 1.81; *p* > 0.2; *F*_interaction_(6,90) = 3.51; *p* < 0.005]. The distribution of biting behavior showed that controls displayed two major bouts of bite delivery, one between min 3 and 6, and another one between min 18 and 21, i.e., toward the end of the stimulation period (**Figure 2C**). MRR stimulation profoundly altered this distribution: bite delivery was frequent in-between stimulations, but significantly less frequent when stimulations were administered (χ^2^ = 31.95; *p* < 0.005). There were no significant differences between the non-stimulated and sham-stimulation periods in controls (χ^2^ = 0.28; *p* > 0.6), but in stimulated mice, non-stimulation and stimulation periods differed significantly (χ^2^ = 5.55; *p* < 0.02) (**Figure 2C**, right-hand panel).

Data suggested a phasic-like effect of MRR stimulation on aggressive behavior. To investigate this issue further we studied the duration of offensive behaviors in bins of 1 min (**Figure 2D**). The time course of offensive behaviors was markedly different in MRR-stimulated mice as compared to controls (**Figure 2D**, left-hand panel) [F_treatment_(1,15) = 0.03; *p* > 0.9; *F*_time_(20,300) = 1.64; *p* < 0.05; *F*_interaction_(20,300) = 1.61; *p* < 0.05]. For clarity, we illustrated this difference by averaging each non-stimulation min that preceded the stimulation periods, as well as each of the 3 min of the stimulation periods (**Figure 2D**, right-hand panel). Stimulations decreased offensive behaviors rather rapidly, e.g., during the first min of their administration and this effect carried over to the next min. Interestingly, however, offense returned to control levels during the third min when stimulation was still administered.

A similar analysis was not performed for bite counts, as their display was sparse, and a min-by-min analysis would have been meaningless. Other behaviors were not affected by stimulations (**Table 1**).

**Table 1:** The effect of MRR optic stimulation on non-social behaviors on day 1.

Stimulation	3 min block							ANOVA statistics

	**1–3**	**4–6**	**7–9**	**10–12**	**13–15**	**16–18**	**19–21**	
*Exploration*		
No	33.2 ± 5.7	58.1 ± 4.4	64.5 ± 3.6	63.2 ± 6.6	70.4 ± 5.0	65.3 ± 7.1	67.4 ± 4.2	*F*_treatment_(1,15) = 0.11; *p* > 0.7 *F*_time_(6,90) = 22.95; *p* < 0.0001 *F*_interaction_(6,90) = 1.33; *p* > 0.7
Yes	24.6 ± 4.2	70.0 ± 4.1	63.8 ± 3.8	71.1 ± 4.4	66.8 ± 5.6	67.9 ± 4.9	67.7 ± 3.1	
*Grooming*		
No	0.9 ± 0.7	3.5 ± 2.4	2.8 ± 1.3	4.7 ± 2.2	2.0 ± 1.3	7.9 ± 3.8	4.1 ± 1.5	*F*_treatment_(1,15) = 0.31; *p* > 0.5 *F*_time_(6,90) = 1.41; *p* > 0.2 *F*_interaction_(6,90) = 0.85; *p* > 0.5
Yes	1.4 ± 0.9	2.3 ± 1.3	4.8 ± 3.5	3.0 ± 1.4	1.2 ± 0.4	2.7 ± 1.1	5.6 ± 2.1	
*Resting*		
No	2.5 ± 2.0	3.4 ± 1.9	4.0 ± 3.2	1.5 ± 0.9	2.7 ± 1.8	4.6 ± 2.9	3.0 ± 1.3	*F*_treatment_(1,15) = 1.57; *p* > 0.2 *F*_time_(6,90) = 0.24; *p* > 0.9 *F*_interaction_(6,90) = 1.02; *p* > 0.4
Yes	2.0 ± 0.9	0.0 ± 0.0	1.4 ± 0.8	2.7 ± 1.8	0.0 ± 0.0	0.0 ± 0.0	2.3 ± 2.3	


*Data (mean ± SEM) show the duration of behaviors expressed in percent of total test time. Sham and real stimulation periods were indicated in gray and blue, respectively. Values showed a significant temporal evolution without significant impact of MRR stimulation.*

Taken together, these findings show that the optogenetic stimulation of the MRR specifically inhibits aggressive behaviors in a phasic-like manner, particularly offense and bite delivery. Non-aggressive social interactions remained unaltered.

### Acute Effects of Intermittent DR Optic Stimulation

In controls, the duration of social behavior followed the same temporal evolution as in the first experiment [*F*_time_(6,72) = 37.56;ughout the encounter [*F*_treatment_(1,12) = 4.66; *p* = 0.05]. The two groups showed small differences before the first stimulation (min 0–3). The two groups showed small differences before the first stimulation (min 0–3). To test whether this affected group differences in later phases of the encounter, we performed a second analysis, in which pre-stimulation and post-stimulation behaviors were evaluated separately. Between min 0–3, differences in social interactions were not significant [*F*(1,12) = 0.82; *p* < 0.4]. By contrast, group differences (expressed as % of min 0–3 values) were significant [*F*_treatment_(1,12) = 6.47; *p* < 0.03; *F*_time_(5,60) = 3.57; *p* < 0.01; *F*_interaction_(5,60) = 0.21; *p* > 0.9]. There was no interaction between the factors, suggesting that DR stimulation had a tonic-like effect. In order to visualize the lack of impact of baseline differences, we showed post-stimulation values as the percentage of pre-stimulation ones in the insert of **Figure 3**. Offensive behavior decreased throughout the encounter [*F*_time_(6,72) = 5.87; *p* < 0.0001] (**Figure 3B**). This was slightly different from the pattern seen in the first experiment, where offense was low in min 0–3, increased between min 3–6 and decreased thereafter (**Figure 2B**). However, a min-by-min presentation of the findings suggests that the patterns of change in controls (i.e., non-stimulated mice) were similar of the two experiments (**Figure 3B**, insert). The figure suggests that offensive aggression was decreased by DR stimulation, but due to large variation the change was not significant [*F*_treatment_(1,12) = 3.09; *p* > 0.2; *F*_interaction_(6,72) = 0.41; *p* > 0.9]. When, however, averages were calculated for the duration of this behavior over the whole encounter (**Figure 3B**, right-hand panel), there was a trend toward decreased aggressiveness in stimulated mice [*F*_treatment_(1,12) = 3.66; *p* = 0.07]. The temporal evolution of bite delivery was also similar to that observed in the first experiment: in controls, two main bouts of bite delivery were identified particularly between min 6–9 and min 18–21 of the encounter (**Figure 3C**). DR stimulation did affect bite counts (Chi square for all time-points = 11.97; *p* < 0.05), but this effect was restricted to min 6–9, e.g., to the 3 min block that followed the first stimulation (Chi square for this time-point = 3.89; *p* < 0.05) (**Figure 3C**).

Non-social behaviors were not affected by DR stimulation (**Table 2**).

**Table 2:** The effect of DR optic stimulation on non-social behaviors on day 1.

Stimulation	3 min block							ANOVA statistics
	**1**	**2**	**3**	**4**	**5**	**6**	**7**	

*Exploration*
No	26.8 ± 6.3	58.6 ± 4.3	70.6 ± 2.8	74.5 ± 6.3	66.7 ± 4.9	68.6 ± 9.3	63.0 ± 7.2	*F*_treatment_(1,12) = 2.09; *p* > 0.1 *F*_time_(6,72) = 17.57; *p* < 0.0001 *F*_interaction_(5,60) = 0.19; *p* > 0.8
Yes	32.7 ± 7.5	66.0 ± 3.5	72.5 ± 3.3	75.4 ± 6.0	73.8 ± 3.4	75.9 ± 5.6	73.2 ± 4.8	
*Grooming*
No	0.2 ± 0.2	3.4 ± 1.6	4.1 ± 2.1	5.2 ± 3.7	6.2 ± 2.2	13.1 ± 5.3	8.4 ± 3.5	*F*_treatment_(1,12) = 2.10; *p* > 0.1 *F*_time_(6,72) = 2.31; *p* < 0.05 *F*_interaction_(5,60) = 1.16; *p* > 0.3
Yes	0.3 ± 0.3	3.6 ± 1.7	2.9 ± 1.4	5.8 ± 2.3	3.2 ± 2.2	3.4 ± 1.6	4.6 ± 1.7	
*Resting*
No	3.0 ± 1.6	1.1 ± 0.9	3.6 ± 1.9	7.0 ± 4.3	4.9 ± 4.7	2.0 ± 2.0	2.3 ± 1.5	*F*_treatment_(1,12) = 0.04; *p* > 0.8 *F*_time_(6,72) = 1.24; *p* > 0.2 *F*_interaction_(5,60) = 0.18; *p* > 0.9
Yes	0.0 ± 0.0	1.9 ± 1.7	4.5 ± 4.5	6.1 ± 4.0	4.3 ± 2.8	3.1 ± 1.8	0.9 ± 0.6	


*Data (mean ± SEM) show the duration of behaviors expressed in percent of total test time. Sham and real stimulation periods were indicated in gray and blue, respectively. Values showed a significant temporal evolution without a significant impact of DR stimulation.*

Taken together, these findings show that DR stimulation increases non-aggressive social interactions, and decreases offensive behaviors at trend level. DR stimulation also abolished the peak in biting behavior observed in controls between 6 and 9 min. Neither of these effects was restricted to the periods of stimulation, suggesting that the DR exerts tonic effects on behavior.

### Carryover Effects of Repeated MRR and DR Optic Stimulation

Subsequent to the first encounter, mice were submitted to three additional ones at 2-day intervals. Optogenetic stimulations were administered according to a roman square design (see Experimental design). As such, all mice had a history of stimulations by the end of the second trial. To identify carryover effects, the actual stimulation status of mice was not considered, because it was hypothesized that carryover effects, if present, would be independent of the ongoing stimulation. Noteworthy, there was no significant interactions between time and stimulation in trials 2–4.

No carryover effects were observed with MRR stimulation. **Figure 4A** presents behavioral differences as compared to the first trial; no statistically significant changes were observed [social behavior: *F*(3,48) = 0.43; *p* > 0.8; offense: *F*(3,48) = 1.11; *p* > 0.4]. The same was true for social behaviors in the case of DR stimulation [*F*(3,24) = 0.38; *p* > 0.8] (**Figure 4B**, left-hand panel).

By contrast, offensive behavior was dependent on the history of DR stimulation (**Figure 4B**, right-h and panel, and **Figure 4C**). As compared to trial 1, offensive behaviors decreased in trials 3 and 4, when all mice had a stimulation history [*F*(3,24) = 6.44; *p* < 0.01]. **Figure 4C** shows that indeed, this effect did not depend on the actual stimulation status of mice. The duration of offensive threats decreased over trials [*F*_trial_(3,20) = 5.98; *p* < 0.01], but no stimulation effects were observed [*F*_stimulation_(1,20) = 0.12; *p* > 0.8], and there was no interaction between these factors [*F*_interaction_(3,20) = 0.57; *p* > 0.7]. Behavioral data obtained in trials 2–4 were shown in more detail in **Tables 3, 4** (for the first trial, see **Figures 1–3**). These tables show that the behavioral effects resulting from stimulation in trial 1 were roughly replicated in subsequent trials, except for the gradual decrease in offense after DR stimulation. No similar decrease was observed after MRR stimulation.

**Table 3:** The effects of median raphe region stimulation on social interactions and offense.

(1) Social interactions										

**Trial**		**3-min blocks**							***Average***	***SEM***

	**Stimulation**	**1**	**2**	**3**	**4**	**5**	**6**	**7**		

2	Yes	44,34	22,78	13,31	20,60	14,67	19,94	12,78	*21,20*	*2,53*
	No	44,71	20,64	15,88	12,42	16,68	7,74	9,14	*18,17*	*2,15*
3	Yes	48,46	14,34	20,40	14,83	16,68	11,94	10,51	*19,59*	*2,19*
	No	44,86	22,95	18,90	15,25	11,74	11,56	10,53	*19,40*	*2,42*
4	Yes	57,28	20,68	20,65	17,19	11,03	10,06	12,65	*21,36*	*3,12*
	No	51,95	25,72	19,54	16,76	20,95	11,75	11,69	*22,62*	*2,22*
*Yes*	*Average*	*51,69*	*19,49*	*17,04*	*16,33*	*14,48*	*13,72*	*11,05*		
	*SEM*	*3,15*	*2,21*	*1,75*	*1,99*	*1,77*	*1,98*	*1,31*		
*No*	*Average*	*48,27*	*22,79*	*17,54*	*15,14*	*16,67*	*9,56*	*10,64*		
	*SEM*	*3,18*	*1,95*	*2,24*	*1,92*	*1,82*	*1,39*	*1,28*		
(2) Offense

**Trial**		**3-min blocks**							***Average***	***SEM***

	Stimulation	**1**	**2**	**3**	**4**	**5**	**6**	**7**		

2	Yes	11.23	4.45	7.30	3.43	4.89	5.86	7.33	*6.36*	*1.08*
	No	14.47	6.92	6.12	7.22	6.51	7.31	4.30	*7.55*	*1.33*
3	Yes	8.72	2.67	4.12	1.80	2.00	4.54	4.41	*4.04*	*0.76*
	No	14.21	8.11	11.30	7.96	5.41	1.81	1.42	*7.17*	*1.34*
4	Yes	10.57	0.03	3.30	9.93	0.04	8.09	0.26	*4.60*	*1.30*
	No	11.82	9.82	3.76	6.00	8.85	6.68	6.18	*7.59*	*1.04*
*Yes*	*Average*	*10.18*	*2.74*	*6.34*	*4.35*	*4.92*	*7.38*	*5.05*		
	*SEM*	*1.68*	*0.66*	*1.15*	*1.29*	*1.50*	*1.94*	*1.23*		
*No*	*Average*	*12.06*	*9.63*	*7.32*	*6.95*	*7.20*	*5.79*	*4.78*		
	*SEM*	*2.23*	*1.55*	*1.57*	*1.39*	*1.68*	*1.57*	*1.26*		


*Data (mean ± SEM) show the duration of behaviors expressed in percent of total test time. Real stimulation periods were indicated in blue. Averages were outlined in bold font. For statistics, see text and **Figure 4**.*

**Table 4:** The effects of dorsal raphe stimulation on social interactions and offense.

(1) Social interactions										

**Trial**		**3-min blocks**							***Average***	***SEM***

	**Stimulation**	**1**	**2**	**3**	**4**	**5**	**6**	**7**		

2	Yes	29,44	13,09	7,13	6,79	19,13	6,52	8,09	***12,89***	*2,36*
	No	32,11	12,77	8,79	5,48	4,72	6,07	4,18	***10,59***	*2,58*
3	Yes	37,34	11,19	10,57	9,63	11,73	6,77	7,03	***13,46***	*2,39*
	No	25,08	11,11	9,33	3,37	12,22	3,11	2,33	***9,51***	*2,08*
4	Yes	29,65	17,08	16,57	8,02	10,94	9,78	13,65	***15,10***	*2,13*
	No	32,74	11,25	8,33	10,42	7,57	8,08	11,02	***12,77***	*1,86*
*yes*	*Average*	***32,66***	***13,53***	***11,34***	***8,29***	***13,71***	***7,60***	***9,33***		
	*SEM*	*3,92*	*3,23*	*2,59*	*1,18*	*2,36*	*2,05*	*1,53*		
*no*	*Average*	***30,42***	***11,76***	***8,77***	***6,70***	***7,80***	***5,99***	***6,16***		
	*SEM*	*4,29*	*2,85*	*2,06*	*1,45*	*1,85*	*1,14*	*1,89*		

(2) Offense

**Trial**		**3-min blocks**							***Average***	***SEM***

	**Stimulation**	**1**	**2**	**3**	**4**	**5**	**6**	**7**		

2	Yes	23,26	32,37	13,28	1,35	13,39	9,41	4,20	***13,90***	*3,57*
	No	29,65	11,32	10,28	6,89	4,32	14,29	4,67	***11,63***	*2,27*
3	Yes	19,97	8,84	11,71	8,74	4,67	1,85	4,61	***8,63***	*1,52*
	No	32,17	9,70	11,24	5,28	13,22	0,00	0,00	***10,23***	*3,00*
4	Yes	27,65	1,83	0,00	1,81	5,94	0,00	0,00	***5,32***	*2,39*
	No	12,59	5,06	0,00	5,02	1,29	1,24	6,08	***4,47***	*1,24*
*Yes*	*Average*	***23,26***	***13,80***	***8,67***	***4,45***	***7,67***	***3,56***	***3,11***		
	*SEM*	*4,10*	*6,36*	*2,14*	*1,81*	*3,01*	*1,90*	*1,43*		
*No*	*Average*	***24,13***	***8,60***	***6,80***	***5,77***	***5,65***	***5,65***	***3,91***		
	*SEM*	*4,88*	*2,52*	*2,20*	*2,56*	*2,68*	*2,48*	*1,93*		


*Data (mean ± SEM) show the duration of behaviors expressed in percent of total test time. Real stimulation periods were indicated in blue. Averages were outlined in bold font. For statistics, see text and **Figure 4**.*

Taken together, these findings show that DR but not MRR stimulations have carryover effects. Particularly, offensive threats decreased in mice with a history of stimulation, and this effect was independent of ongoing stimulations. Considering that offense was affected by DR stimulation only at trend level in trial 1, and that aggression levels decreased in trials 3 and 4 irrespective to current stimulation status, one can hypothesize that the mechanisms underlying this phenomenon are different from those that underlie the acute effects of DR stimulation.

### Neurotransmitter Release in the Prefrontal Cortex After MRR and DR Optic Stimulation

The neurochemical consequences of raphe stimulations were studied in the prefrontal cortex, an area deeply involved in the control of aggression and social behavior in general. Importantly, the particularities of stimulations were similar to those employed in the behavioral studies, albeit mice were anesthetized this time.

The prefrontal release of all three, serotonin, glutamate, and GABA were increased after the optogenetic stimulation of raphe nuclei [*serotonin*: *F*_time_(7,119) = 7.06; *p* < 0.01; *glutamate*: *F*_time_(7,119) = 2.56; *p* = 0.01; *GABA*: *F*_time_(7,119) = 4.14; *p* < 0.01]. Moreover, at the termination of the experiment 100 mM KCl was able to increase neurotransmitter release remarkably in all animals confirming that the cells remained alive and reactive (*p* < 0.01 comparing the last fraction to all others except stimulated ones) (**Figure 5**, columns). However, the neurochemical consequences of MRR or DR stimulation largely depended on the stimulated brainstem area [*serotonin*: *F*_time^∗^group_(14,119) = 2.44; *p* < 0.01; *glutamate*: *F*_time^∗^group_(14,119) = 1.82; *p* < 0.05; *GABA*: *F*_time^∗^group_(14,119) = 1.62; *p* = 0.082]. As compared to baseline, the extracellular release of serotonin was increased during the optogenetic stimulation of both the MRR and DR (**Figure 5A**). Note that the samples contained a microdialysate of 30 min, whereas stimulations lasted only 3 min. Consequently, release induced by stimulation was considerably diluted, which may explain the relatively low levels of serotonin in the dialysate. The temporal evolution of the release was, however, rather different with the two nuclei [*F*_groups_(2,17) = 9.68; *p* < 0.01]. The increase vanished relatively rapidly when the MRR was stimulated., whereas DR stimulation induced a long lasting increase in release: prefrontal serotonin levels were higher 2 h after the last stimulation as compared to controls.

Glutamate release was increased only in mice stimulated in their MRR. DR stimulation had no similar effect (**Figure 5B**). Note that in contrast to serotonin, the increase in glutamate release was observed after a considerable delay, but at the same time the effect was lasting, as it was observed 1h after the first stimulation. GABA release increased immediately after the first stimulation as with serotonin release, but was transient in both groups [*F*_groups_(2,17) = 2.03; *p* > 0.1] (**Figure 5C**).

We also investigated the release of dopamine and noradrenaline in the prefrontal cortex; stimulations affected neither (data not shown).

These findings show that the stimulation of the MRR and DR show some similarities as it regards their neurochemical consequences in the prefrontal cortex, but also show important differences. The impact of stimulations on GABA release was similar in the groups. By contrast, glutamate release was induced by MRR stimulation only, whereas the release of serotonin -albeit present in both groups- was transient with MRR stimulation, and surprisingly long-lasting with DR stimulations.

## Discussion

### Main Findings

The dorsal and median raphe affected social behavior and aggression differently in our study. MRR stimulations decreased aggression in a phasic-like manner. Effects were restricted to the stimulation periods, and vanished in the non-stimulation periods that separated stimulations. No effects on social behaviors were observed. By contrast, the DR stimulation rapidly promoted social behaviors, but in a tonic fashion. Effects were present during both the stimulation and non-stimulation periods. Aggressive behaviors were marginally diminished by DR stimulation in the first trial, but repeated stimulations administered over 8 days considerably decreased aggression suggesting that repeated DR stimulations have slowly developing effects.

The effects of MRR and DR stimulation on neurotransmitter release were markedly different in the prefrontal cortex, a major site of aggression control. MRR stimulation increased serotonin release relatively rapidly, but transiently, and induced a major and more durable increase in glutamate release. By contrast, DR stimulation had no effect on glutamate release, but persistently increased prefrontal levels of serotonin. Release remained higher than the baseline long after stimulations halted. Effects on GABA release were transient with both nuclei.

### Raphe Nuclei and Serotonin

Ample evidence demonstrates that the neurochemical properties of raphe neurons are heterogenous: about their half or more are non-serotonergic (depending on the study; Moore, 1980). Glutamatergic and GABAergic neurons are significant components of both raphe nuclei, some studies suggesting that they are more numerous than serotonergic ones (*DR*: (Gamrani et al., 1979; Nanopoulos et al., 1982; Commons, 2009; Jackson et al., 2009); *MRR*: (Allers and Sharp, 2003; Varga et al., 2009; Sos et al., 2017); moreover, disparate studies suggest that the share of serotonergic neurons is below 10% in the median raphe (Sos et al., 2017). In addition, serotonergic neurons often co-express (sometimes several) other neurotransmitters, suggesting that even serotonergic neurons release non-serotonergic neurotransmitters (Kachidian et al., 1991; Shutoh et al., 2008; Gagnon and Parent, 2014; Sos et al., 2017). As such, behavioral effects obtained by the stimulation of raphe nuclei are not necessarily attributable to serotonin.

Although the existence of long-range GABAergic neurones was repeatedly suggested (Melzer et al., 2012; Caputi et al., 2013; Lee et al., 2014), no earlier publication confirmed that the axon terminals of raphe GABA neurons can reach the prefrontal cortex, the GABA response to stimulation was likely secondary to the release of other neurotransmitters, e.g., serotonin or glutamate which responded to raphe stimulation in our study. It is worth to note, however, that a large share of raphe neurons seems to be neither glutamatergic, GABAergic nor serotonergic (Sos et al., 2017). Such neurons may express other neurotransmitters, e.g., dopamine (Jahanshahi et al., 2013). Albeit the connectivity of some non-serotonergic raphe neurons is poorly known, one cannot rule out that they contributed to the behavioral effects observed here, as all three serotonin, glutamate and dopamine contribute to the control of aggression by the prefrontal cortex (Takahashi et al., 2011; Hwa et al., 2015; Tielbeek et al., 2016). The particular roles of these raphe mechanisms can be investigated only by neuron type-specific expression of channelrhodopsin, e.g., by the use of CRE mice. A differential study of such subsystems may be the target of subsequent research.

### Differential Role of Raphe Nuclei in Aggression: Comparisons With Earlier Studies

While the inhibition of aggression by the dorsal raphe is well-established (Pucilowski and Kostowski, 1983; Takahashi and Miczek, 2014; Miczek et al., 2015), the role of the median raphe is more controversial. Early studies provided negative results; e.g., DR lesions lastingly promoted aggressive behavior, whereas MRR lesions were without effect (Jacobs and Cohen, 1976). In a similar fashion, the stimulation of the dorsal raphe did, whereas the stimulation of the median raphe did not inhibit aggression in a study involving muricide (Pucilowski and Kostowski, 1981). It occurs that more subtle manipulations also emphasize the role of the DR over those of the MRR. E.g., the activation of GABA_B_ receptors in the DR but not in the MRR promoted the display of escalated aggression (Takahashi et al., 2010). Other studies did find a role for MRR in aggression control; e.g., the 5,7-dihydroxytryptamine-mediated destruction of the MRR decreased submissiveness in rats and elicited behaviors indicative of aggressive arousal albeit not aggression *per se* (File et al., 1979). In another study, however, counterintuitive effects of MRR downregulation were observed. The 5-HT1A receptor agonist 8-OH-DPAT, an activator of somatodendritic autoreceptors, decreased maternal aggression when microinjected into the MRR of female rats (De Almeida and Lucion, 1997). Thus, the downregulation of MRR serotonin neurotransmission achieved by negative feedback decreased rather than increased aggressiveness.

We suggest that such controversial findings may at least be partly explained by the phasic-like effects of MRR neurotransmission on aggression as revealed by the present study. Such effects may easily be overlooked in studies using different experimental approaches, as the anti-aggressive effects of MRR stimulation seem to vanish rather rapidly. In our study, offense decreased in the first two, but returned to control levels during the third min of stimulation (**Figure 2D**). Earlier findings corroborated with our release studies may even suggest that the anti-aggressive effects of MRR stimulations may be reversed over time. It was shown that a short pulse of serotonin is likely to induce inhibition in the cortex, whereas the prolonged presence of serotonin may result in excitation (Zhou and Hablitz, 1999). In line with these observations, MRR stimulation increased serotonin and GABA release within 15 min in the prefrontal cortex, but these effects disappeared upon repeated stimulations to give raise to a large increase in glutamate release (**Figure 5**). One can tentatively hypothesize that this change in the neurochemical consequences of stimulations may have reversed their behavioral effects if stimulations were more durable. The complex neurochemical effects of MRR stimulation may at least partly explain the controversial findings briefly reviewed above.

### Limitations

As a first attempt to differentiate the roles of the two raphe nuclei in sociability and aggression by optogenetic techniques, our experiments have limitations, which need to be addressed in future studies. We investigated neurotransmitter release only in the prefrontal cortex, and in anesthetized animals. There are several other key regions in the circuitry that controls aggression, and neurotransmitter release may be influenced by anesthesia, albeit controls were also anesthetized. Nevertheless, our microdialysis study revealed two important aspects of raphe function. One was technical: the study showed that the stimulation of the DR and MRR induces serotonin release in areas involved in aggression control. Serotonin was outlined here because in contrast to glutamate and GABA, its release cannot be attributed to local neurons. The second important conclusion of this study was that the stimulation of the DR and MRR elicits substantially different neurochemical responses (at least in the prefrontal cortex). The differential neurochemical consequences of stimulations can be attributed to the particularities of the two raphe nuclei rather than to anesthesia.

The second limitation of the study relates to differences in the temporal resolution of the behavioral and the neurochemical experiment. Behaviors were investigated in bins of 3 min, whereas neurotransmitter release was studied in samples taken at 30 min intervals due to technical reasons. Fraction No. 4 reflected fast responses, because stimulation was started just 15 min before this fraction was collected. Fraction No.5 indicated habituation/serotonin depletion due to repeated stimulations, whereas subsequent fractions indicated prolonged effects. Based on findings, one can confidently assume that the serotonin and GABA response occurred shortly after stimulation, whereas the glutamate response was slower. Yet, the next sample was taken with a delay, thus, it is impossible to evaluate how slow the glutamate response was. A more rapid sampling and detection methodology can overcome this deficiency in the future. Nevertheless, our findings may provide a preliminary clue on the mechanisms underlying the behavioral effects. For instance, the phasic-like effects of MRR stimulation are unlikely to be mediated by glutamatergic neurotransmission, because the behavioral response appears to be faster than the glutamatergic one. On the other hand, the tonic-like behavioral effects of DR stimulation may be due to serotonin release, as this was the only persistent neurochemical response observed in the microdialysis study. One cannot rule out that blue light *per se* had effects, as sham stimulations were in fact no stimulations. Yet, our studies performed in parallel showed that blue light *per se* has no measurable effects on social behavior (Biro et al., 2018). The issue naturally needs further experimentation.

Albeit not necessarily a limitation, we mention here that we failed to observe those rapid behavioral effects of raphe stimulation that were described in several laboratories, including ours (Ohmura et al., 2014; Balazsfi et al., 2017; Correia et al., 2017). In these studies, the stimulation of the DR or the MRR rapidly suppressed locomotion, increased anxiety, or resulted in the emergence of conditioned fear. Effects usually developed within seconds except for conditioned fear, but even in this study (Balazsfi et al., 2017) rapid effects on locomotion were evident when mice were stimulated in the MRR. No locomotion effects were observed in the social interaction test in the present experiments. It is unlikely that the reason was technical, as the aforementioned study of ours was performed under entirely similar conditions than this one. One can tentatively hypothesize that the environment has a decisive impact on the consequences of raphe stimulation. E.g., effects induced in non-social testing environments (Ohmura et al., 2014; Balazsfi et al., 2017; Correia et al., 2017) may be overruled or changed in social contexts (this study).

## Conclusion

Our findings suggest that the raphe nuclei provide several ways to control the social behaviors. They inhibit aggression in a phasic-like manner, while increasing amiable interactions tonically. Both effects are rapid, but have a different time-course. The two effects dissociate anatomically: the phasic-like control of aggression can be attributed to the MRR, whereas the tonic control of social behaviors to the DR. The latter also seems to exert a slowly developing anti-aggressive effect, which can be expressed independently of actual DR activation. The differential roles of the two raphe nuclei are likely explained by their differential neurotransmission profiles in target areas.

Understanding the role of serotonin in aggression requires information on both the anatomical source of serotonergic inputs at various release sites, and the elucidation of the interactions between various neurotransmitter systems located within the raphe nuclei. It has been suggested that MRR and DR projections differ in their sensitivity toward pharmacological agents (Hensler, 2006). If true, a better understanding of the separate roles of raphe nuclei in aggression may help understanding controversial findings with the available agents and may also help designing novel treatment strategies.

## Author Contributions

DB and DZ microinjected the virus carrier and implanted the optic fibers; they also performed behavioral and microdialysis experiments, and contributed to designing the study, as well as to the analysis and interpretation of findings. KD together with GN and CC checked the optic fiber and microdialysis probe placements and virus infection by immunocytochemistry. CM, ZV, and ÁN contributed to the scoring of behaviors, whereas MB and BS studied neurotransmitters in dialysates obtained from the prefrontal cortex. JH contributed to the designing of the study, the interpretation of findings, and wrote the first draft of the manuscript.

## Conflict of Interest Statement

The authors declare that the research was conducted in the absence of any commercial or financial relationships that could be construed as a potential conflict of interest.

## Footnotes

1 https://solomoncoder.com/
